# Supplementary material for: Asynchronous magnetic resonance elastography: Shear wave speed reconstruction using noise correlation of incoherent waves
Source: Magn Reson Med. 2022 Oct 27;89(3):990–1001. doi: 10.1002/mrm.29502 (PMC9792433; doi:10.1002/mrm.29502)
Supplement: Supplementary file 1 — DATA S1 MATLAB code used to generate the numerical simulations of Figure 1 [file MRM-89-990-s002.zip › k-Wave/helpfiles/writeGrid.html]

writeGrid :: Functions (k-Wave)


# writeGrid

Write grid and PML properties to a k-Wave HDF5 file.

## Syntax

```
writeGrid(filename, grid_size, grid_spacing, pml_size, pml_alpha, Nt, dt, c_ref)
```

## Description

`writeGrid` creates and writes the wavenumber grids and PML variables required by the k-Wave C++ code to the HDF5 file specified by the user.

List of parameters that are written:

- `Nx`
- `Nx`
- `Ny`
- `Nz`
- `Nt`
- `dt`
- `dx`
- `dy`
- `dz`
- `c_ref`
- `pml_x_alpha`
- `pml_y_alpha`
- `pml_z_alpha`
- `pml_x_size`
- `pml_y_size`
- `pml_z_size`

## Inputs

|  |  |
| --- | --- |
| `filename` | name of HDF5 file to write matrix to |
| `grid_size` | `[Nx, Ny, Nz]` |
| `grid_spacing` | `[dx, dy, dz]` |
| `pml_size` | `[pml_x_size, pml_y_size, pml_z_size]` |
| `pml_alpha` | `[pml_x_alpha, pml_y_alpha, pml_z_alpha]` |
| `Nt` | number of time points |
| `dt` | time step |
| `c_ref` | scalar sound speed used in the k-space operator and to define the pml variables |

## See Also

`h5write`, `writeAttributes`, `writeFlags`, `writeMatrix`
